# Expression of periostin and podoplanin in canine testicular tumours

**DOI:** 10.1186/s12917-025-05176-y

**Published:** 2025-12-09

**Authors:** Rafał Ciaputa, Izabela Janus-Ziółkowska, Kacper Żebrowski, Marcin Nowak, Stanisław Dzimira, Aleksandra Piotrowska, Katarzyna Ratajczak-Wielgomas, Małgorzata Kandefer-Gola, Valeria Grieco

**Affiliations:** 1 Department of Pathology, Division of Pathomorphology and Veterinary Forensics, Faculty of Veterinary Medicine, Wrocław University of Environmental and Life Sciences, C.K. Norwida 31, Wrocław, 50-375 Poland; 2 Division of Histology and Embryology, Department of Human Morphology and Embryology, Wrocław Medical University, Wrocław, 50-368 Poland; 3 Department of Veterinary Medicine and Animal Science, University of Milan, via dell’Università 6, Lodi, 26900 Italy

**Keywords:** Testis, Cancer, Oncology, Pathology, Ki-67, Protein, Dog, Immunohistochemistry

## Abstract

**Background:**

Periostin (POSTN) and podoplanin (PDPN) are both proteins playing an important role in humans in the diagnosis and study of many malignant tumours. POSTN affects the regulation of intracellular signalling pathways involving protein kinases PI3-K/Akt and focal adhesion kinase, whereas PDPN increases fibroblast migration and affects the structure of the endothelium; both proteins participate in carcinogenesis, by increasing cell migration and intensifying angiogenesis. In veterinary medicine, their expression has been demonstrated in only a few types of neoplasms, however their presence has not been investigated in canine testicular tumours which are common in dogs and represent a reliable animal model for their human counterparts, due to their structure and biological behaviour. In the present retrospective study, 186 canine testicular tumours namely 61 Leydig cell tumours, 64 Sertoli cell tumours and 61 seminomas and 10 normal canine testicles were immunohistochemically tested for expression of POSTN, PDPN and Ki67 antigens. The polymerase chain reaction (PCR) analysis was also performed on stored frozen samples representative of the three tumour types: 10 Leydig cell tumours, 9 Sertoli cell tumours and 9 seminomas, which confirmed the presence of both proteins.

**Results:**

Normal testes were negative for both POSTN and PDPN, whereas 27 (44.3%) Leydig cell tumours, 54 (84.4%) Sertoli cell tumours and 56 (91.8%) seminomas were cytoplasmically positive for POSTN and 23 (37.7%), 49 (77.6%) and 44 (72.1%) were cytoplasmically positive for PDPN respectively. The intensity of POSTN and PDPN immunohistochemical reaction was stronger in Sertoli cell tumours and seminomas than in Leydig cell tumours. In addition, Ki67 antigen expression for both POSTN and PDPN correlated significantly with the number of positive cells and the intensity of the reaction in seminomas and Sertoli cell tumours.

**Conclusions:**

The results showed the absence of expression of POSTN and PDPN in normal canine testes and their expression in neoplastic ones, suggesting a role for these proteins in the carcinogenesis of the testis and encouraging further studies, in particular, on seminomas and Sertoli cell tumours.

## Background

The steady increase in the incidence of cancer in animals and humans justifies the search for new methods of pathomorphological diagnosis and accurate approaches, which may be useful for the characterisation of tumours and the assessment of patient prognosis. In addition, the tumours diagnosed are increasingly occurring in younger patients and are often malignant, making the study of carcinogenesis processes and the proteins involved very important [1–3]. In humans, proteins that are absent or present at very low levels in normal cells, but are characteristically expressed by corresponding neoplastic cells are of particular interest in carcinogenesis, as they may be useful in diagnosis and/or represent a therapeutic target [4–17]. In veterinary medicine, these proteins contribute to better diagnosis and treatment of cancer in patients and may indicate potential animal models for studying relevant cancers in humans with POSTN and PDPN being among such proteins [4–17].

POSTN, a 93 kDa glycoprotein composed of 836 amino acids, was first identified in a mouse osteoblast cell line (MC3T3-E1) and named ‘osteoblast-specific factor-2’ [10, 18]. POSTN influences the regulation of intracellular signalling pathways involving the protein kinases PI3-K/Akt and focal adhesion kinase (FAK) and is thus involved in carcinogenesis and, in addition, increases cell migration and promotes angiogenesis [13, 14, 18]. It has also been shown to play a significant role in the ontogenetic process by influencing epithelial-mesenchymal transition (EMT) and extracellular matrix (ECM) remodelling [13, 14, 18]. In humans, POSTN expression has been detected not only in cancer-associated fibroblasts (CAFs), but also in the cells of multiple tumour types, including breast, ovarian, lung, colon and pancreatic cancers [4, 6, 18–20]. Very little is known about the role of POSTN in neoplastic processes within animals, and there are only a few published reports. To date, POSTN expression has been demonstrated in canine tumours such as mammary carcinoma, bladder carcinoma, osteosarcoma and squamous cell carcinoma of the skin [18, 21–25], whereas there is no information on its expression in canine testicular tumours.

PDPN, an O-glycosylated sialoglycoprotein consisting of 162 transmembrane amino acids, was first identified in mouse osteoblast cell lines (MC3T-E1) and in ras-transformed MC3T3 cells [15, 16, 26]. Based on the identification of this protein in rat podocytes, it was named podoplanin [17, 26]. Like POSTN, PDPN is used in humans in the diagnosis of a variety of tumours, and its presence has also been found in CAFs and tumour cells, for example in breast cancer, oesophageal cancer, and lung adenocarcinoma [8, 9, 27–29]. It has also been shown to increase the migration of human fibroblasts and affect the structure of endothelial cells, which is similar to the role of POSTN in carcinogenesis [26, 30]. In veterinary medicine, PDPN, similar to POSTN, has been recognised and described in some lesions such as mammary carcinoma, keratinising squamous cell carcinoma, melanoma or colon adenocarcinoma [26, 31–33], however there is no information about its presence in testicular neoplasms.

Thus, due to and taking into account the high incidence of testicular tumours in dogs and the fact that they have been proposed as a model for the study of their human counterparts, the aim of the present study was to analyse the expression of POSTN and PDPN, and their possible correlation with the Ki67 antigen - a standardised prognostic factor for canine testicular tumours [34, 35] - in three different tumour types: Leydig cell tumour (LCT), Sertoli cell tumour (SCT) and seminoma (SEM**)**.

## Methods

### Materials

For the present retrospective study, cases of canine testicular tumours collected between 2016 and 2021 were selected and retrieved from the archive of the Department of Pathology at the Wroclaw University of Environmental and Life Sciences. The selection criteria were that the breed and age of the dog were recorded in the archive database and that histological slides and related blocks referring to a complete longitudinal section of the tumour were available. Additionally, paraffin blocks of 10 normal canine testis (complying with the same selection criteria) were included in the study as controls.

All normal and neoplastic testicular samples were from adult dogs undergoing orchiectomy, whether it is of a routine or therapeutic nature, or had been collected during post-mortem examination. No tumours were intentionally induced in the animals, and no animals were surgically treated or killed for the study. Testicular tumours, belonging to dogs of different ages (Table 1), were classified according to the World Health Organization’s (WHO) classification of tumours of the genital system in domestic animals [36]. For Sertoli cell tumours and seminomas, the tumour growth pattern was recorded as either intratubular or diffuse. Interstitial (Leydig) cell tumours were classified as solid–diffuse, cystic–vascular, or pseudoadenomatous (Table 2) [36]. There were 186/200 tumours within the selection criteria and 227 related paraffin blocks. Tumours were distributed as follows: 61 LCTs, 64 SCTs and 61 SEMs.

**Table 1:** Types and number of testicular tumours diagnosed in dogs of different ages

Age	LCT *n* = 61	SCT *n* = 64	SEM *n* = 61
6	0	0	3
7	2	2	7
8	2	3	8
9	11	15	2
10	9	15	7
11	12	12	8
12	8	11	11
13	12	1	7
14	1	3	7
15	0	2	1
16	0	0	0
17	4	0	0

*LCT* Leydig cell tumor, *SCT* Sertoli cell tumor, *SEM* Seminoma, *n* number of cases

**Table 2:** Number and histological features of testicular tumours according to the WHO classification

Growth pattern	LCT *n* = 61	SCT *n* = 64	SEM *n* = 61
Cystic-vascular (LCT)	34		
Solid (LCT)	17		
Psuedoadenomatous (LCT)	10		
Intratubular (SCT - SEM)		23	17
Diffuse (SCT - SEM)		41	44

*LCT* Leydig cell tumor, *SCT* Sertoli cell tumor, *SEM* Seminoma, *n* number cases

In addition, previously collected samples of unfixed frozen testicular tumours were selected for real-time polymerase chain reaction (RT-qPCR) analysis, including 10 Leydig cell tumours, 9 Sertoli cell tumours and 9 seminomas, which were preserved in RNAlaterTM Soln. (Cat. No. AM7024, Thermo Fisher Scientific, Inc. Foster City, CA, USA) and frozen at −80 °C.

### Immunohistochemistry

Serial 3 µm sections were obtained from the paraffin blocks of both normal and neoplastic testes and immunohistochemical reactions were performed using Autostainer Link48 (Agilent, Santa Clara, CA, USA). Slides were deparaffined and epitope retrieval was conducted by heating the slides in EnVision FLEX Target Retrieval Solution (Agilent) (97°C, 20 min; pH 9 for POSTN and PDPN and pH 6 for Ki-67) using PTLink Platform (Agilent, Santa Clara, CA, USA). First, endogenous peroxidase activity was blocked by 5 min exposure to EnVision FLEX Peroxidase-Blocking Reagent (Agilent, Santa Clara, CA, USA). Afterwards, slides were incubated for 20 min in RT with primary antibodies. Antibodies targeting POSTN, PDPN and Ki67, rabbit polyclonal anti-Periostin antibody (1:100, cat.no. NBP1-82472, Novus Biologicals, Littleton, CO, USA), monoclonal mouse anti-Podoplanin (clone D2-40) antibody (ready-to-use, cat. no. IR072, Agilent, Santa Clara, CA, USA) and monoclonal mouse anti-Ki-67 antibody (ready-to-use, IR626, Agilent, Santa Clara, CA, USA), had already been used in previous studies [18, 21, 26, 37]. The similarity between dog and human tested proteins was verified by the basic local alignment search tool (BLAST). Secondary antibodies were applied (20 min, RT). The reactions were visualised using substrate for horseradish peroxidase (DAB – 3,3’-diaminobenzidine), with incubation for 10 min at RT. After, all the sections were counterstained with FLEX Hematoxylin (Agilent, Santa Clara, CA, USA) for 5 min at RT, dehydrated in graded ethanol alcohol (70%, 96%, 99,8%) and xylene. Finally, the slides were mounted in Mounting Medium (Agilent, Santa Clara, CA, USA). A negative control was obtained by replacing the primary antibody with Diluent (Agilent, Santa Clara, CA, USA).

Immunohistochemical results of the positive cytoplasmic reaction for POST and PDPN were assessed by two experienced pathologists and were scored using two independent semiquantitative scores as shown in Table 3. The A-score considered the percentage of positive cells, whereas the B-score considered the intensity of immunohistochemical labelling. The Ki-67 value was expressed as the percentage of positively stained cells for nuclear reaction for the whole tissue. The value was calculated by counting 1000 cells per section and, as in previous papers, scored based on the percentage of positive cells: 0–5% = no reaction (negative − 0 points), 6–25% = low reaction (1 point), 26–50% = moderate reaction (2 points), above 50% = high reaction (3 points) (400× magnification**)** [37, 38].

**Table 3:** Semiquantitative scores used to evaluate immunohistochemical expression of POSTN and PDPN in tumour and normal testicular cells

A - Percentage of cells positive	Points
Negative	0
1–40%	1
41–60%	2
61–80%	3
81–90%	4
91–100%	5

B - Intensity	Points
Weak	1
Moderate	2
Intense	3

Microscopic photographs of the testicular sections underwent computer-aided image analysis using a computer coupled with an Olympus BX53 optical microscope (Olympus, Japan) equipped with a digital Olympus ColorView IIIu camera (Olympus, Japan). The measurements were taken using Cell^A software (Olympus Soft Imaging Solution GmbH, Germany).

### Reverse transcription-quantitative PCR (RT-qPCR)

Total RNA was extracted from the studied tissues with the use of the RNeasy Mini Kit (Qiagen Hilden, Germany), according to the manufacturer’s protocol. In order to eliminate genomic DNA contamination, on-column DNase digestion was performed using the RNase-Free DNase Set (Qiagen). First-strand cDNA was synthesised with the High-Capacity cDNA Reverse Transcription Kit (Applied Biosystems; Thermo Fisher Scientific, Inc.). RT-qPCR was performed using 7500 Real-Time PCR System (Applied Biosystems, Foster City, CA, USA, RRID: SCR_014596), primers and probes of the TaqMan system (Applied Biosystems, Foster City, CA, USA). Cf02680557_m1 for POSTN, cf4931159_m1 for ACTB (Applied Biosystem) were the primers and TaqMan probes were used in the study. All reactions were obtained under the following conditions: activation of polymerase at 50 °C for 2 min, initial denaturation at 94 °C for 10 min, 40 cycles of denaturation at 94 °C for 15 s, followed by annealing and elongation at 60 °C for 1 min. The results were standardised in relation to the expression of the reference gene of β-actin.

### Statistical analysis

Statistical analysis was performed using Statistica 13.3 (TIBCO Software Inc.) and appropriately selected statistical tests. Normality of data was tested using the Shapiro-Wilk test. The comparison of POSTN and PDPN expressions present in the three different types of tumour and LCTs subtypes were performed using Kruskal-Wallis ANOVA analysis with post-hoc Dunn’s test. The comparison of POSTN and PDPN expressions present in the SEMs and SCTs subtypes were performed using Mann-Whitney U analysis. Correlations were tested using the Spearman rank correlation test. The level of significance was assumed for *p* ≤ 0.05.

## Results

### Immunohistochemistry

**Fig. 1:**
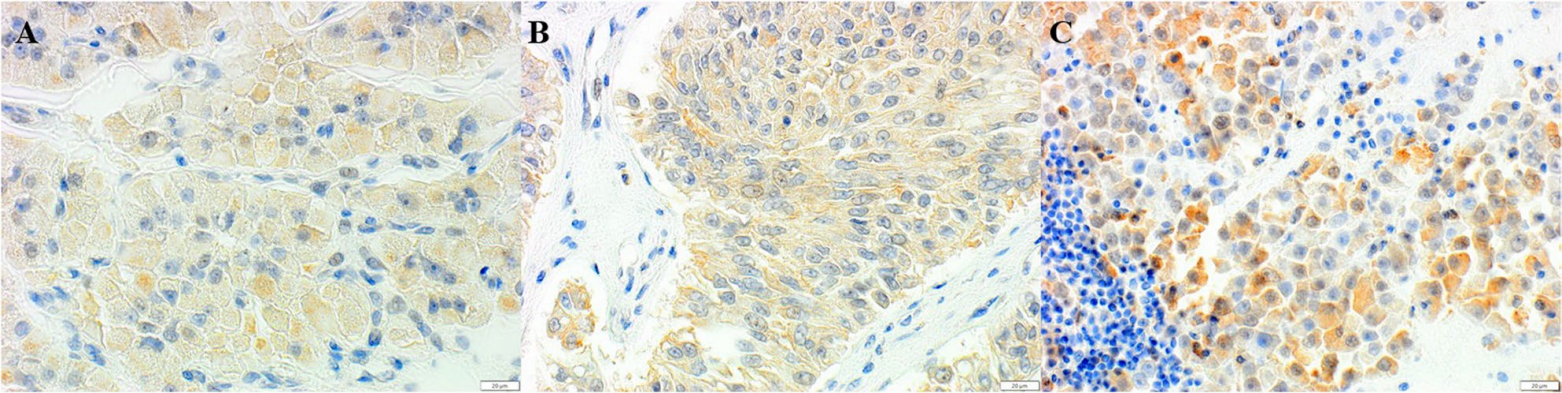
Immunohistochemistry for POSTN. Positive cytoplasmic reaction in neoplastic cells of LCTs (**A**), SCTs (**B**), SEM (**C**) (400X). DAB immunohistochemistry, Mayer’s Hematoxylin counterstain, BAR 20 μm

**Fig. 2:**
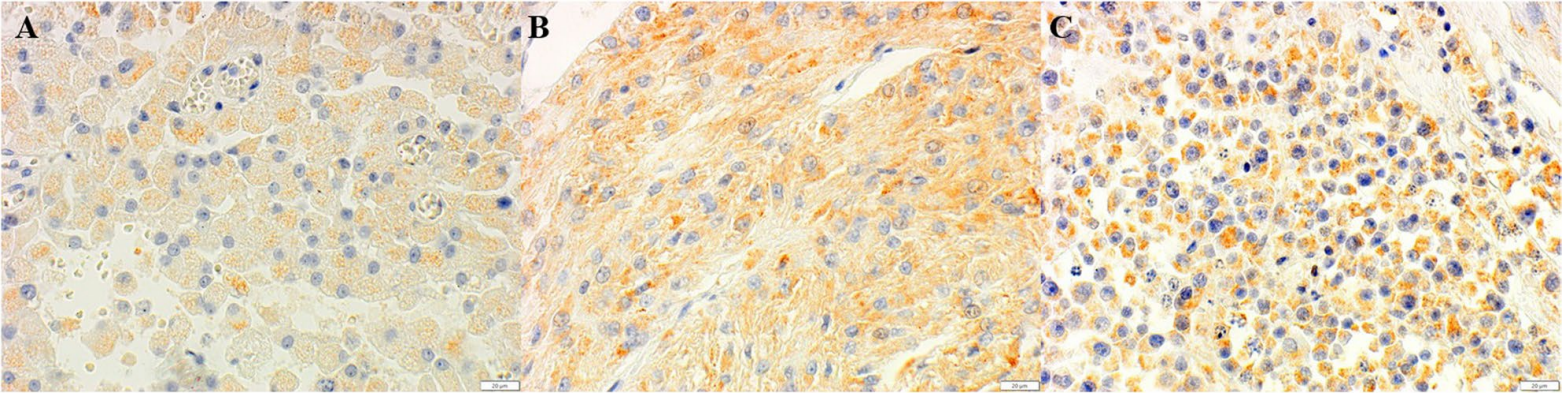
Immunohistochemistry for PDPN. Positive cytoplasmic reaction of in neoplastic cells of LCTs (**A**), SCTs (**B**), and SEMs (**C**) (400X). DAB immunohistochemistry, Mayer’s Hematoxylin counterstain, BAR 20μm

**Fig. 3:**
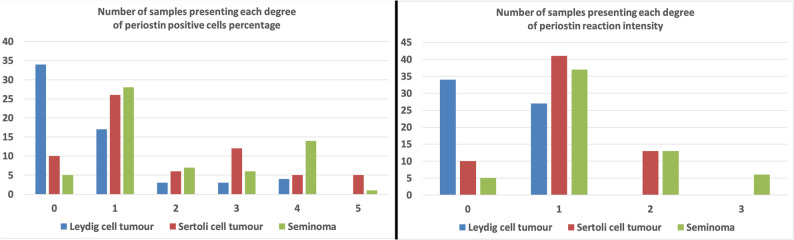
The number of samples exhibiting the expression of POSTN in LCTs, SCTs and SEMs

**Fig. 4:**
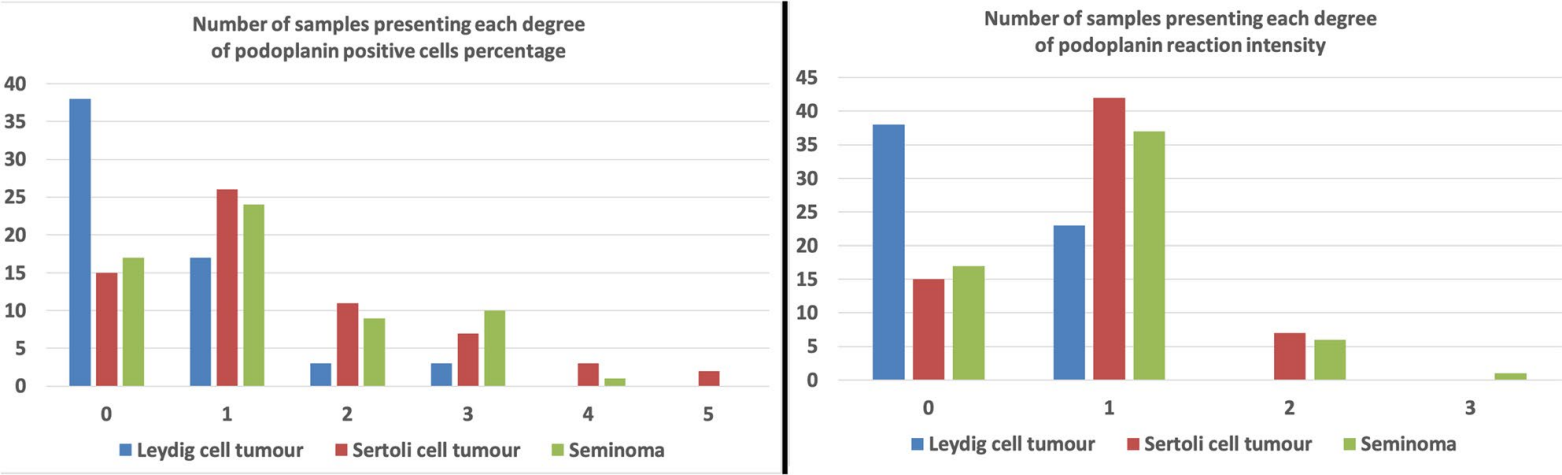
The number of samples exhibiting the particular expression of PDPN in LCTs, SCTs and SEMs

In the assessment of immunohistochemical results, agreement between the two pathologists was reached for each sample in terms of indicating both the score for the percentage range of positive cells and the intensity of the reaction. All normal testes were negative for both POSTN and PDPN. Of the 186 canine testicular tumours, 137 were positive for POSTN and 116 for PDPN. The positive signal, which was consistently cytoplasmic (Figs. 1 and 2), was detected in a variable percentage of neoplastic cells, as was the intensity of the immunohistochemical reaction among cases (Figs. 3 and 4). Results are summarised in Table 4.

**Table 4:** Immunohistochemical results. Percentage of positive cells and intensity of reaction for POSTN and PDPN in the 186 canine testicular tumours analysed

Scores		LCT *n* = 61		SCT *n* = 64		SEM *n* = 61	
A. Percentage of positive cells	Points	POSTN	PDPN	POSTN	PDPN	POSTN	PDPN
Negative	0	34	38	10	15	5	17
1–40%	1	17	17	26	26	28	24
41–60%	2	3	3	6	11	7	9
61–80%	3	3	3	12	7	6	10
81–90%	4	4	-	5	3	14	1
91–100%	5	-	-	5	2	1	-

B. Intensity	Points						
Weak	1	27	23	41	42	37	37
Moderate	2	-	-	13	7	13	6
Intense	3	-	-	-	-	6	1

*LCT* Leydig cell tumor, *SCT* Sertoli cell tumor, *SEM* Seminoma, *n* number of cases

#### Leydig cell tumours

POSTN reaction was negative in 34/61 (55.7%) of tumours evaluated. In 17/27 of positive cases (27.9%), POSTN was expressed in 1–40% of neoplastic cells, in 3 cases (4.9%) positive cells ranged between 41 and 60% while, in 3 and 4 cases, the positive range was 61–80% and 81–90% respectively. The intensity of the POSTN reaction was weak in all the 27 positive tumours (44.3%).

PDPN immunohistochemical reaction was negative in 38/61 of cases (62.3%) and positive in 23 cases (37.7%). In 17 (27.9%) cases the percentage of positive cells scored 1 point, while in 3 cases (4.9%) it scored 2 points, and in another 3 cases (4.9%) it scored 3 points. As for POSTN, the intensity of the reaction for PDPN was weak in all positive LCTs.

The Ki-67 protein reaction was absent (0 points) in 24 out of 61 cases (39.4%), while it was low (6–25% − 1 point) in 34 cases (55.7%) and moderate (26–50% − 2 points) in 3 (4.9%) cases.

#### Sertoli cell tumours

POSTN was not expressed in 10/64 of cases (15.6% tumours). In the 54 positive tumours, a variable percentage of positive cells was detected. In 26 (40.6%) cases, 1–40% of neoplastic cells were positive, 6 tumours (9.4%) had 41–60% of positive cells, in 12 (18.8%) tumours, the percentage of positive neoplastic cells was 61–80%, in the next 5 (7.8%) tumours, positive cells accounted for 81–90%, and in the remaining 5 (7.8%) tumours, positive cells accounted for 91–100%.The intensity of expression of POSTN protein in Sertoli cells in 41 tumours, i.e. 64.1%, was weak and moderate in 13 tumours (20.3%).

PDPN reaction was negative in 15 cases, i.e. (23.4%) and positive in the remaining 49 cases (76.6%). In 26 of these 49 cases (40.6%), the number of cells gave a score of 1 point. 11 tumours (17.2%) scored 2, 7 tumours (11.0%) scored 3 points, 3 tumours (4.7%) scored 4 points, and 2 tumours (3.1%) scored 5 points. PDPN expression was weak in 42 tumours (65.6%) and moderate in 7 tumours (11.0%).

In 64 SCTs, Ki-67 protein was absent (0 points) in 2 cases (3.1%), low (6–25%, 1 point) in 39 cases (60.9%), moderate (26–50%, 2 points) in 17 cases (26.6%), and high (> 50%, 3 points) in 6 cases (9.4%).

#### Seminomas

POSTN reaction was negative in only 5/61 of tumours (8.2%). In 28 out of the 56 (45.9%) positive cases, the percentage of positive cells was scored 1 point, whereas 7 (11.5%) cases scored 2 points, 6 (9.8%) scored 3 points, and 14 (23.0%) scored 4 points. There was only 1 (1.6%) case where all neoplastic cells were positive (5 points). The intensity of POSTN protein expression in SEMs was weak in 37 tumours (60.70%), moderate in 13 (21.30%) and high in 6 case (9.80%).

PDPN reaction was negative in 17 cases (27.9%). Among the 44 positive cases, 24 (39.3%) scored 1 point, 9 (14.8%) scored 2 points, 10 (16.4%) scored 3 points, and 1 (1.6%) scored 4 points. PDPN expression was weak in 37 tumours (60.7%), moderate in 6 (9.8%) tumours and intense in 1 (1.6%) tumours.

Protein Ki-67 expression, detected in all seminomas, was low (6–25% −1 points) in 17/61 of cases, moderate (26–50% − 2 points) in 23 cases (37.7%) and high (3 points) in 21 cases (34.4%).

### Real-time PCR

The expression of the POSTN and PDPN genes at the mRNA level was found in 100% (*n* = 28) of the analysed testicular tumours. The same tumours were also immunohistochemically positive for both POSTN and PDPN.

### Statistics

Statistical analysis did not reveal any significant relationship between the expression of POSTN and PDPN, either at the mRNA level or at the immunohistochemical expression level (Spearman correlation test; *p* > 0.05).

**Fig. 5:**
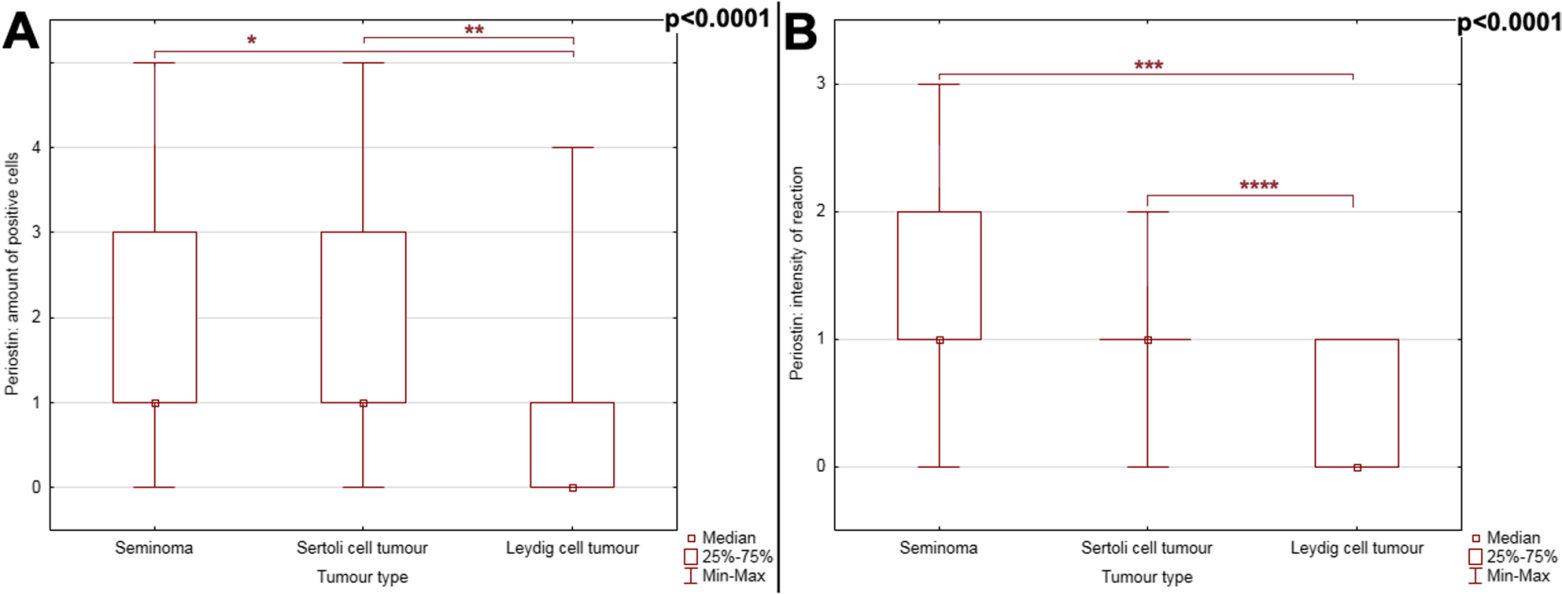
POSTN immunohistochemical reaction in the three types of testicular neoplasms considered. Difference between tumours has been noted in: (**A**) the number of positive cells and (**B**), the intensity of the reaction (*p* < 0.0001). P-value obtained by the Kruskal-Wallis ANOVA analysis with post-hoc Dunn test: * *p* < 0.0001; ** *p* < 0.0001; *** *p* < 0.0001; **** *p* < 0.0001

**Fig. 6:**
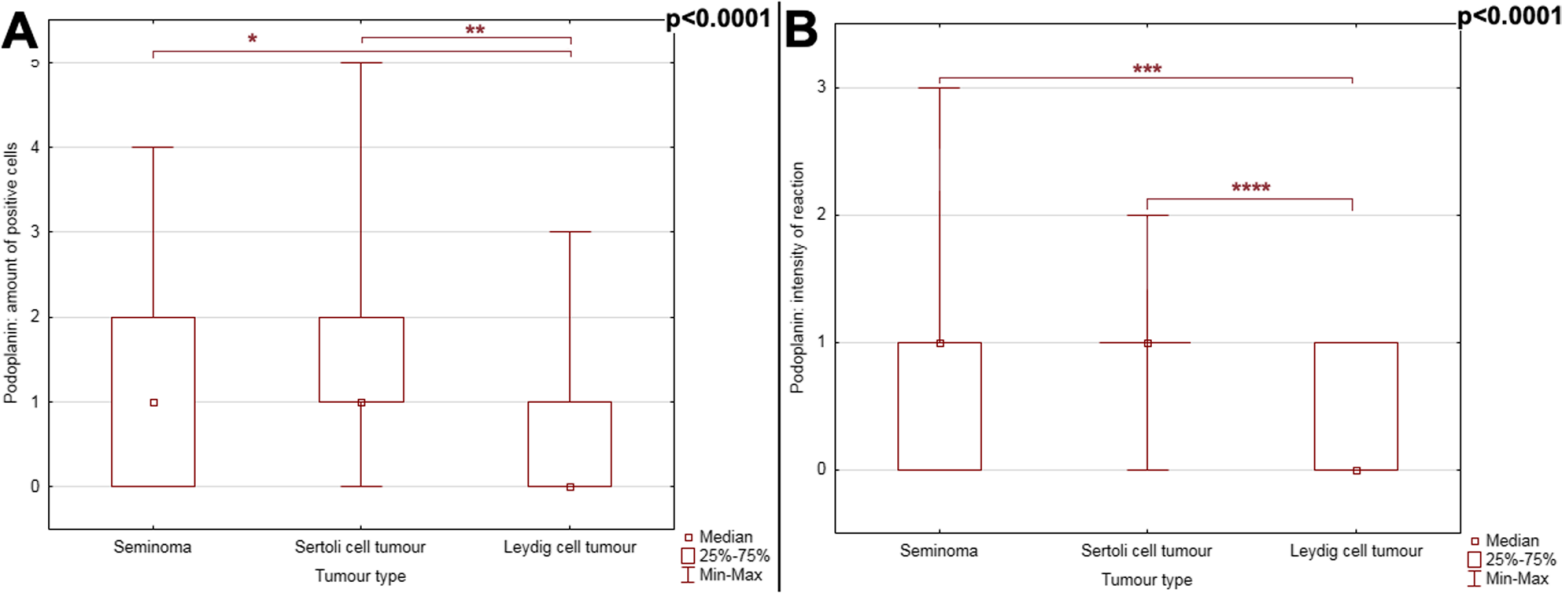
PDPN immunohistochemical reaction in the three types of testicular neoplasms considered. Difference between tumours has been noted in: (**A**) the number of positive cells and (**B**), the intensity of the reaction (*p* < 0.0001). P-value obtained by the Kruskal-Wallis ANOVA analysis with post-hoc Dunn test: * *p* < 0.0001; ** *p* < 0.0001; *** *p* < 0.0001; **** *p* < 0.0001

The expression of both POSTN and PDPN was statistically significantly lower in LCTs than in SCTs and SEMs, both in terms of the number of positive cells and the intensity of the immunohistochemical reaction (Figs. 5 and 6) (*p* < 0.0001). There were no statistically significant differences between the subtypes of individual tumour types (cystic-vascular, solid and pseudoadenomatous for LCTs, intratubular and diffuse for either SCTs or SEMs; *p* > 0.05 for all comparisons). Significant correlations between the expression of POSTN and PDPN and the number of positive cells and/or the intensity of the reaction were observed within each tumour type, as presented in the following paragraphs.

In LCTs, a positive correlation was found between the intensity of the POSTN immunohistochemical reaction and the number of positive cells (*r* = 0.96; *p* < 0.0001), the intensity of the reaction and Ki-67 (*r* = 0.66; *p* < 0.0001), and the number of positive cells and Ki67 (*r* = 0.68; *p* < 0.0001). For PDPN expression, similar to POSTN, a positive correlation was found between the intensity of the reaction and the number of positive cells (*r* = 0.98; *p* < 0.0001), the intensity of the reaction and Ki-67 (*r* = 0.48; *p* < 0.0001), and the number of positive cells and Ki67 (*r* = 0.66; *p* < 0.0001).

In SCTs, there was a positive correlation between the number of positive cells and the intensity of POSTN expression in neoplastic cells (*r* = 0.71; *p* < 0.0001). Similarly, Ki67 correlated positively with the number of positive cells (*r* = 0.36; *p* = 0.004) and the intensity of POSTN expression (*r* = 0.34; *p* = 0.005). Positive correlations between the number of positive cells and staining intensity (*r* = 0.82; *p* < 0.0001) were also observed for PDPN in SCTs.

In SEMs, a statistically significant positive correlation was found between the intensity of the POSTN reaction and the number of positive cells (*r* = 0.77; *p* < 0.0001) and between the intensity of the reaction and Ki-67 (*r* = 0.28; *p* = 0.03). In the case of PDPN, a positive correlation was found between the intensity of the reaction and the number of positive cells (*r* = 0.86; *p* < 0.0001).

## Discussion

Scientific reports [5–7, 18, 21, 25–27] have shown that POSTN and PDPN proteins play an important role in the carcinogenesis of various types of neoplasms in both humans and animals. However, with regard to testicular cancer, there are no reports of POSTN expression in human and canine testicles, and there are only two studies on PDPN in humans [39, 40].

Over the last few decades, the number of cases of testicular neoplasia among both men and dogs has steadily increased [1–3]. Some reports suggest that dogs may be a good animal model for the study of human testicular tumours because they share similar living conditions with humans, and are the only species in which spontaneous testicular tumours are common and morphologically similar to their human counterparts [35, 41].

Both POSTN and PDPN were absent in normal testes, but immunohistochemically present in most tumours, clearly indicating their role in testicular carcinogenesis.

Furthermore, the results of the immunohistochemical tests were validated and corroborated by the expression of the POSTN and PDPN genes at the mRNA level, which was detected in 100% (*n* = 28) of the tumours analyzed using the Real-time PCR method. Although all 28 of these samples were also positive in immunohistochemical tests, a percentage of the 186 tumours examined were negative for one or both proteins. This finding may be explained by several biological and technical factors. Firstly, the detection of mRNA for genes such as POSTN and PDPN indicates active transcription but does not confirm the presence of corresponding functional proteins. Mechanisms of translational repression, including silencing via microRNAs and other post-transcriptional control mechanisms, may inhibit protein synthesis despite detectable mRNA levels. Moreover, translated proteins may undergo rapid degradation or fail to accumulate to levels detectable by immunohistochemistry. Real-time PCR is inherently more sensitive than immunohistochemical techniques, which rely on antibody affinity and tissue availability. Furthermore, abnormal protein localization or post-translational modifications may hinder antigen recognition, further limiting immunohistochemical detectability [42–46].

These results obtained in canine species are very interesting because they provide relevant information regarding POSTN, that has not yet been investigated in human testes, and are also fully consistent with what has been reported in the literature regarding PDPN in humans [39, 40]. Indeed, despite the lack of data on POSTN, PDPN was also not expressed in normal adult human testes [40]. However, it was observed in the foetal testis, particularly in gonocytes, the early germ cells, and in the Sertoli cells, which then lose this expression during maturation [40]. The reappearance of PDPN expression in testicular tumours, observed in humans and also in dogs within this study, suggests that the protein, whose role is not yet fully understood, may be involved in the process of cell dedifferentiation that is part of carcinogenesis.

Analysing POSTN and PDPN expression in neoplastic cells of testicular tumours, we observed that their expression was significantly lower in LCTs than in SCTs and SEMs, both in percentage of positive cells and intensity. This finding corroborates the fact that LCTs in dogs, as in humans, are the less aggressive, with a better prognosis forms of testicular tumours [36, 47, 48].

Conversely, in SCTs and SEMs, which may be more aggressive than LCTs [36, 47, 48], the number of negative cases was lower, and the percentage of positive cells was higher, as was the intensity of expression of both proteins. However, it should be noted that in each tumour type, including LCTs, a significant correlation was observed between the expression of POSTN and PDPN and the number of positive cells and/or the intensity of the reaction. This suggests that both proteins are involved in the process of tumorigenesis.

In addition, to better understand the role of POSTN and PDPN in cancer development, similar to our previous work and that of other authors [18, 21, 26, 27, 49], it was performed an analysis of the correlation of the expression of these two proteins with the expression of the Ki67 antigen. This protein has been routinely used in the diagnosis of cancers in both humans and animals for many years and is applied as a prognostic marker since its expression correlates with cell proliferation [27, 50, 51]. With regard to Ki67 expression, the results of the present study are consistent with those of previous studies and showed both a higher number of positive cases and a higher number of positive cells in SEMs compared to SCTs and an even lower number in LCTs [37, 52].Moreover, it has been demonstrated that, although it was weakest in LCTs, a positive correlation between the expression of POSTN and PDPN and the Ki67 antigen was present in all types of testicular tumours studied.

Upregulation of both POSTN and PDPN has been observed in human and canine mammary cancers indicating that for these tumours, in women as in dogs, both proteins as well as Ki67 may have prognostic significance in the course of neoplastic disease [18, 26, 27, 53, 54]. Similarly, the results obtained in the present study suggest the involvement of these proteins in testicular carcinogensis processes within dogs. This could also confirm their differential participation in tumours that could be of a potentially more aggressive character, such as SCT and SEM, compared to LCT.

The upregulation of the POSTN and PDPN genes observed in canine testicular tumours raises important questions about the fundamental regulatory mechanisms involved. Studies of other malignancies suggest that increased expression of these genes may result from alternative splicing processes, epigenetic modifications such as promoter hypomethylation, or increased protein stabilisation. Additionally, the dysregulation of signalling pathways and transcription activators may contribute to their abnormal expression in tumour tissues [42–46]. Incorporating such mechanistic insights into future studies could enhance the biological significance of the present study’s findings and encourage further research into these pathways in testicular tumours in dogs and humans.

## Conclusion

The expression of POSTN and PDPN has been demonstrated for the first time in canine spontaneous testicular tumours, indicating their participation in the process of testicular carcinogenesis in dogs, as was already suggested for PDPN in human testicular tumours. The higher expression of POSTN and PDPN observed in SCTs and SEMs, compared to LCTs, may reflect the different characteristics of testicular tumours in the dog and could have prognostic and therapeutic implications that encourage further investigation involving a larger number of samples.

## Data Availability

The datasets used and/or analysed during the current study are available from the corresponding author upon reasonable request.

## Abbreviations

POSTN: Periostin
PDPN: Podoplanin
PCR: Polymerase chain reaction
RT-qPCR: Real–time polymerase chain reaction
FAK: Focal adhesion kinase
WHO: World Health Organization
LCT: Leydig cell tumour
SCT: Sertoli cell tumour
SEM: Seminoma
EMT: Epithelial–mesenchymal transition
ECM: Extracellular matrix
CAF: Cancer–associated fibroblast
